# Inhibition of Sevoflurane Postconditioning Against Cerebral Ischemia Reperfusion-Induced Oxidative Injury in Rats

**DOI:** 10.3390/molecules17010341

**Published:** 2011-12-30

**Authors:** Yan Zhang, Fu-Geng Zhang, Chun Meng, Shou-Yuan Tian, Ya-Xin Wang, Wei Zhao, Jun Chen, Xiu-Shan Zhang, Yu Liang, Shi-Dong Zhang, Yan-Jie Xing

**Affiliations:** 1 Department of Anesthesiology, Tianjin Huanhu Hospital, Tianjin 300060, China; Email: yzhangtjhh13@sina.com (Y.Z.); yaxinw_007@yahoo.cn (Y.-X.W.); junchen_hhyy@qq.com (J.C.); xiushan_zhang@sina.cn (X.-S.Z.); yliang75@yahoo.cn (Y.L.); 2 Department of Pharmacy, Tianjin Huanhu Hospital, Tianjin 300060, China; Email: fugengzh16@126.com; 3 Department of Anesthesiology, The First Hospital Affiliated Shanxi Medical University, Taiyuan, Shanxi 030001, China; Email: shouyuansx@yeah.net; 4 Metabolic Disease Hospital, Tianjin Medical University, Tianjin 300070, China; 5 Key Lab of Hormones and Development, Ministry of Health and Tianjin, Tianjin 300070, China; Email: weizhao_tj@163.com; 6 Department of Anesthesiology, Jinghai Hospital, Tianjin 300060, China; Email: shidongzhang_jh@tom.com; 7 Department of Anesthesiology, Tangshan City Worker Hospital, Tianjin 300060, China; Email: yanjie_xing@tom.com

**Keywords:** antioxidant, immunity, rats, cerebral ischemia reperfusion, TNF-α, sevoflurane postconditioning

## Abstract

The volatile anesthetic sevoflurane is capable of inducing preconditioning and postconditioning effects in the brain. In this study, we investigated the effects of sevoflurane postconditioning on antioxidant and immunity indexes in cerebral ischemia reperfusion (CIR) rats. Rats were randomly assigned to five separate experimental groups I–V. In the sham group (I), rats were subjected to the same surgery procedures except for occlusion of the middle cerebral artery and exposed to 1.0 MAC sevoflurane 90 min after surgery for 30 min. IR control rats (group II) were subjected to middle cerebral artery occlusion (MCAO) for 90 min and exposed to O_2_ for 30 min at the beginning of reperfusion. Sevoflurane 0.5, 1.0 and 1.5 groups (III, IV, V) were all subjected to MCAO for 90 min, but at the beginning of reperfusion exposed to 0.5 MAC, 1.0 MAC or 1.5 MAC sevoflurane for 30 min, respectively. Results showed that sevoflurane postconditioning can decrease serum tumor necrosis factor-alpha (TNF-α), interleukin-1 beta (IL-1β), nitric oxide (NO), nitric oxide synthase (NOS) and increase serum interleukin-10 (IL-10) levels in cerebral ischemia reperfusion rats. In addition, sevoflurane postconditioning can still decrease blood lipid, malondialdehyde (MDA) levels, infarct volume and increase antioxidant enzymes activities, normal pyramidal neurons density in cerebral ischemia reperfusion rats. It can be concluded that sevoflurane postconditioning may decrease blood and brain oxidative injury and enhance immunity indexes in cerebral ischemia reperfusion rats.

## 1. Introduction

Oxidation, which provides energy to biological processes, is essential to organisms. However, reactive oxygen species, which are continuously generated when oxygen is reduced by single electrons *in vivo*, can cause extensive damage to lipids, proteins, DNA and other components of organisms. Reactive oxygen species have been implicated in the pathogenesis of oxidative stress-related diseases, such as stroke, cancer, cardiovascular diseases, hypertension, ageing, *etc*. [1,2,3,4]. Antioxidants are significant substances which can protect the body from oxidative damage [5].

In middle cerebral artery occlusion (MCAO)-induced cerebral ischemia, various biochemical events occur that cause intracellular calcium accumulation, depolarization, excessive release of excitatory amino acids, especially glutamate, and inhibition of protein synthesis [6,7]. Cerebral ischemia is one of the leading causes for several neurological deficits and death [8] and the causative mechanism suggested explaining this phenomenon is the involvement of reactive oxygen species (ROS) and oxidative stress [9,10]. ROS clearly have a role in gene activation [11] and subsequent events leading to ischemic cell death [12].

Extensive research has been done to find effective strategies and drugs to ameliorate or prevent brain ischemia and reperfusion injury. Many anesthetics such as propofol [13,14] and dexmedetomidine [15,16] have neuroprotective effects. Especially volatile anesthetics, such as sevoflurane and isoflurane, have been shown to mimic the most potent protective mechanisms and exert direct neuroprotective effects *in vitro* and *in vivo* [17,18]. Yu *et al.* [19] examined the role of the phosphatidylinositol-3-kinase (PI3K)/Akt pathway in anesthetic postconditioning and determined whether PI3K/Akt signaling modulates the expression of pro- and antiapoptotic proteins in sevoflurane postconditioning. Activation of PI3K and modulation of the expression of pro- and antiapoptotic proteins may play an important role in sevoflurane-induced myocardial protection. Sevoflurane-induced preconditioning protects against cerebral ischemic neuronal damage in rats [20]. Yao *et al.* [21] investigated the cardioprotection induced by sevoflurane postconditioning (SpostC) in rat hearts *in vitro*, and the respective role of phosphatidylinositol-3-kinase (PI3K), extracellular signal-regulated kinase 1 and 2 (ERK 1/2), mitochondrial KATP channels (mitoKATP) and mitochondrial permeability transition pore (mPTP), by selectively inhibiting PI3K, ERK 1/2, mitoKATP, with LY294002 (LY), PD98059 (PD), 5-hydroxydecanoate (5-HD) and by directly opening of mPTP with atractyloside (ATR), respectively. In the present experiment, we examine the protective effect of sevoflurane postconditioning against oxidative injury in cerebral ischemia reperfusion rats.

## 2. Results and Discussion

### 2.1. Effects of Sevoflurane Postconditioning on Serum TNF-α, IL-10 and IL-1β Concentrations

TNF-α and IL-1β are two important inflammatory cytokines and play an important role in pro-inflammation after brain IR injury. Reperfusion can stimulate the transcription and expression of TNF-α and IL-1β. Both cytokines can exert chemotactic effect mediating the rolling, adherence, and penetration of neutrophils toward endothelial cells and promoting the migration, proliferation and infiltration of peripheral inflammatory cells toward ischemic tissues [22]. As shown in Table 1, the serum TNF-α, and IL-1β concentrations were significantly (*p* < 0.01) higher in the cerebral ischemia reperfusion (CIR) rats (group II), whereas serum IL-10 level was markedly lower when compared with the sham rats. Sevoflurane postconditioning concentration-dependently significantly (*p* < 0.01) decreased the serum TNF-α, and IL-1β concentrations and increase serum IL-10 level in CIR rats (IR + sevoflurane) when compared with the CIR rats (group II). Tumor necrosis factor-α (TNF-α) is a pleiotropic cytokine suspected to enhance or deter cellular survival through activation of receptor-mediated signal transduction. The level of TNF-α in human brain becomes elevated after cerebral infarction [23] and appears sequentially in the infarct core and peri-infarct areas before expression in tissue within the unaffected hemisphere [24]. Elevated levels of TNF-α have been observed consistently in serum [25,26,27] and in cerebrospinal fluid [26,27] after acute ischemic stroke. In animal models of cerebral ischemia, high levels of TNF-α have been found after global [28,29] and focal [30] ischemic injury. IL-1β is an important mediator triggering the immune response and inflammation and can be secreted by neurons, astrocytes, oligodendrocytes and endothelial cells. As an inflammatory mediator and an immune-derived cytokines, IL-1β cannot only promote the activation of B cells and T cells by cooperating with other cytokines, but stimulate the proliferation of astrocytes and the secretion of TNF-α, IL-6, nervous growth factor (NGF), granulocyte colony-stimulating factor (GCSF) and NO by astrocytes [31]. Our work indicates that sevoflurane postconditioning can reduce the brain IR-induced inflammation in rats.

**Table 1:** Effects of sevoflurane postconditioning on serum TNF-α, IL-10 and IL-1β concentrations.

Group	TNF-α (ng/mL)	IL-1β (ng/L)	IL-10 (ng/L)
I	1.95 ± 0.11	10.65 ± 1.07	42.06 ± 3.08
II	4.65 ± 0.27 ^b^	19.66 ± 1.43 ^b^	21.52 ± 1.37 ^b^
III	3.06 ± 0.24 ^d^	17.03 ± 1.49 ^c^	29.29 ± 1.33 ^d^
IV	2.66 ± 0.29 ^d^	14.72 ± 1.35 ^d^	33.03 ± 1.97 ^d^
V	1.99 ± 0.2 ^d^	12.18 ± 1.22 ^d^	38.58 ± 1.85 ^d^

^b^* p* < 0.01, compared with group I; ^c^* p* < 0.05, ^d^* p* < 0.01, compared with group II.

### 2.2. Effects of Sevoflurane Postconditioning on Serum NO and NOS Concentrations

NO is an important messenger molecule the body and the effector molecule for neurotransmission, vasorelaxation, regulate nerve function of endogenous media, and the nervous system close to physiological and pathological processes [32], and NOS is the rate-limiting enzyme NO biosynthesis. Studies have reported that antioxidant agents can decrease the expression of ischemia-induced NOS that reduce NO synthesis, thus reducing ischemic brain injury [33]. As shown in Table 2, the serum NO, and NOS concentrations were significantly higher in the CIR rats (group II) when compared with the sham rats. Sevoflurane postconditioning concentration-dependently significantly (*p* < 0.01) decreased the serum NO, and NOS concentrations in CIR rats (CIR + sevoflurane) when compared with the CIR rats (group II). This indicates that sevoflurane postconditioning can reduce ischemic brain injury by decreasing NOS activities and NO levels.

**Table 2:** Effects of sevoflurane postconditioning on serum NO and NOS concentrations.

Group	NO (μmol/L)	NOS (U/mL)
I	21.57 ± 2.07	22.18 ± 1.43
II	40.62 ± 2.31 ^b^	51.43 ± 0.96 ^b^
III	35.15 ± 1.99 ^c^	39.09 ± 1.37 ^d^
IV	29.07 ± 1.26 ^d^	29.04 ± 1.54 ^d^
V	21.36 ± 1.65 ^d^	20.13 ± 1.22 ^d^

^b^* p* < 0.01, compared with group I; ^c^* p* < 0.05, ^d^* p* < 0.01, compared with group II.

### 2.3. Effects of Sevoflurane Postconditioning on Serum Lipids Levels

Brain ischemia induces the release of excitatory amino acids, with subsequent receptor activation leading to calcium influx, metabolic and electrophysiological dysfunction, and oxidative stress (including lipid peroxidation) [34]. Subsequent reperfusion worsens this oxidative stress, potentiating ischemic injury [35]. The concentration of total cholesterol (TC), triacylglycerol (TG) and low density lipoprotein cholesterol (LDL-c) in the plasma of sham and CIR rats is described in Table 3. In CIR rats, plasma TC, TG and LDL-c levels were significantly (*p* < 0.01) increased, whereas plasma high density lipoprotein cholesterol (HDL-c) level was significantly (*p* < 0.01) decreased compared to sham group (I). Sevoflurane postconditioning concentration-dependently significantly (*p* < 0.01) decreased the concentration of TC, TG and LDL-c, and increased the level of HDL-c in group (III-V) compared to CIR group (II). In addition, sevoflurane postconditioning concentration-dependently significantly (*p* < 0.01) reverse the CIR-induced increased the LDL/HDL compared to CIR group (II). In our study, sevoflurane postconditioning improved the LDL/HDL ratio—A finding in keeping with the findings of others. 

**Table 3:** Effects of sevoflurane postconditioning on serum lipids levels.

Group	TC (mmol/L)	TG (mmol/L)	HDL-c (mmol/L)	LDL-c (mmol/L)	LDL/HDL
I	2.92 ± 0.2	0.74 ± 0.05	1.63 ± 0.12	1.02 ± 0.08	0.64 ± 0.04
II	5.84 ± 0.26 ^b^	2.03 ± 0.13 ^b^	0.72 ± 0.08 ^b^	5.21 ± 0.21 ^b^	7.09 ± 0.32 ^b^
III	5.17 ± 0.31 ^c^	1.75 ± 0.11 ^c^	1.03 ± 0.05 ^d^	4.52 ± 0.16 ^c^	4.37 ± 0.18 ^d^
IV	4.53 ± 0.19 ^d^	1.33 ± 0.09 ^d^	1.46 ± 0.08 ^d^	3.62 ± 0.18 ^d^	2.47 ± 0.13 ^d^
V	3.27 ± 0.22 ^d^	0.94 ± 0.07 ^d^	1.55 ± 0.09 ^d^	2.37 ± 0.11 ^d^	1.53 ± 0.09 ^d^

^b^* p* < 0.01, compared with group I; ^c^* p* < 0.05, ^d^* p* < 0.01, compared with group II.

### 2.4. Effects of Sevoflurane Postconditioning on Serum and Brain MDA, Reduced Glutathione (GSH) Concentrations

Oxidative stress is one of the primary factors that exacerbate damage caused by cerebral ischemia [36]. The physiological mechanisms underlying cerebral ischemic injury resulting from increased production of pro-oxidants as well as decreased production of antioxidant defenses have been extensively studied. Although oxygen radicals, by virtue of their reactivity, can injure neurons and other brain cells directly, increased evidence has pointed to the role of redox signaling of oxygen radicals [37]. Such redox signaling targets mitochondrial cytochrome c release, DNA repair enzymes, and transcriptional factor nuclear factor-κB, which might lead to neuronal damage. Free radicals attack lipid membranes, and peroxidative propagation with neurodegeneration occurs by the consecutive production of other oxygen radical species. Several experimental studies indicate that this cascade of reactions induced by ischemia followed by recirculation causes membrane disintegration and irreversible energy failure, leading to the aggravation of brain edema and loss of neuronal functions [38,39]. Superoxide and hydroxyl radical are potent in producing destruction of the cell membrane by inducing lipid peroxidation. The brain is particularly vulnerable to oxidative stress injury because of its high rate of oxidative metabolic activity and intense production of reactive oxygen species metabolites, and its high content of polyunsaturated fatty acids, relatively low antioxidant capacity [40]. In accordance with the increases in ROS, the brain MDA level was significantly increased, indicating the presence of enhanced lipid peroxidation due to IR injury, while the levels of tissue glutathione declined, demonstrating the depletion of the antioxidant pool. Several studies have demonstrated that IR in the brain is associated with lipid peroxidation, which is an autocatalytic mechanism leading to the oxidative destruction of cellular membranes [41,42]. Glutathione, on the other hand, is an important constituent of intracellular protective mechanisms against various noxious stimuli including oxidative stress [43]. Because of their exposed sulfhydryl groups, non-protein sulphydryls bind a variety of electrophilic radicals and metabolites that may be damaging to cells. It has been proposed that antioxidants, which maintain the concentration of reduced GSH, may restore the cellular defense mechanisms, block lipid peroxidation and thus protect against the oxidative tissue damage [44,45].

Table 4 shows the levels of MDA and GSH in blood and brain tissues were found to be increased in group II rats. Sevoflurane postconditioning concentration-dependently significantly (*p* < 0.01) decreased blood and brain MDA and increased GSH concentrations in group III-V rats when compared with CIR control (group II).

**Table 4:** Effects of sevoflurane postconditioning on serum and brain MDA, GSH concentrations.

Group	MDA		GSH	
	Blood (nmol/mL)	Brain (nmol/g prot)	Blood (nmol/mL)	Brain (nmol/mg protein)
I	4.21 ± 0.21	3.15 ± 0.15	43.98 ± 3.08	58.32 ± 2.31
II	8.53 ± 0.37 ^b^	7.04 ± 0.43 ^b^	22.17 ± 1.43 ^b^	20.61 ± 1.26 ^b^
III	7.02 ± 0.42 ^c^	5.82 ± 0.22 ^d^	29.51 ± 1.24 ^d^	32.51 ± 1.32 ^d^
IV	5.97 ± 0.22 ^d^	5.01 ± 0.27 ^d^	35.66 ± 1.75 ^d^	41.28 ± 1.85 ^d^
V	4.88 ± 0.28 ^d^	4.22 ± 0.17 ^d^	40.71 ± 1.95 ^d^	59.03 ± 2.08 ^d^

^b^* p* < 0.01, compared with group I; ^c^* p* < 0.05, ^d^* p* < 0.01, compared with group II.

### 2.5. Effects of Sevoflurane Postconditioning on Serum and Brain SOD, CAT, GSH-Px and GR Activities

The enzymes superoxide dismutase (SOD), catalase (CAT), and glutathione peroxidase (GSH-Px) are the best-known components of the biological protective system [46,47,48,49]. It is recognized that these enzymes may be important protectors against lipid peroxidation and damage due to free radicals after the onset of neuronal ischemia. In the present experiment, the significant (*p* < 0.01) decreases in SOD, CAT, GSH-Px and glutathione reductase (GR) activities in blood and brain was observed in group II rats when compared with group I rats (Table 5). The present study shows that elevation of MDA and depletion of protective enzymes (SOD, CAT, and GSH-Px) in ischemic reperfused brain are in agreement with earlier reports [50]. Sevoflurane postconditioning concentration-dependently significantly (*p* < 0.01) increased blood and brain SOD, CAT, GSH-Px and GR activities in group III-V rats when compared with CIR control (group II). This results are in agreement with previous work [51]. This indicated that sevoflurane postconditioning can enhance antioxidant enzyme activities and decrease oxidative injury in cerebral ischemia reperfusion rats.

**Table 5:** Effects of sevoflurane postconditioning on serum and brain SOD, CAT, GSH-Px and GR activities.

**Group**	**SOD**		**CAT**	
	**Serum (U/mL)**	**Brain** **(U/mg)**	**Serum (U/mL)**	**Brain** **(U/mg)**
I	276.5 ± 16.3	303.1 ± 22.9	39.11 ± 1.54	33.12 ± 1.84
II	132.1 ± 9.6 ^b^	137.4 ± 10.7 ^b^	15.03 ± 1.11 ^b^	12.84 ± 1.02 ^b^
III	176.4 ± 8.6 ^d^	199.2 ± 12.4 ^d^	19.99 ± 2.09 ^d^	21.87 ± 1.15 ^d^
IV	220.6 ± 11.6 ^d^	274.8 ± 17.3 ^d^	24.08 ± 1.57 ^d^	28.44 ± 1.36 ^d^
V	281.5 ± 20.5 ^d^	314.1 ± 19.2 ^d^	34.17 ± 1.72 ^d^	32.17 ± 1.83 ^d^
**Group**	**GSH-Px**		**GR**	
	**Serum (U/mL)**	**Brain** **(U/mg)**	**Serum (U/mL)**	**Brain** **(U/mg)**
I	52.19 ± 2.54	63.19 ± 2.98	29.07 ± 1.54	32.18 ± 1.76
II	22.15 ± 1.54 ^b^	27.51 ± 1.06 ^b^	12.16 ± 1.06 ^b^	15.27 ± 1.13 ^b^
III	32.97 ± 1.97 ^d^	37.82 ± 1.44 ^d^	19.03 ± 1.17 ^d^	21.54 ± 1.08 ^d^
IV	40.63 ± 2.61 ^d^	47.18 ± 2.31 ^d^	24.01 ± 1.32 ^d^	29.41 ± 1.57 ^d^
V	49.76 ± 2.88 ^d^	58.29 ± 2.28 ^d^	32.18 ± 1.69 ^d^	34.29 ± 1.39 ^d^

Values are given as mean ± SD from six rats in each group. ^b^* p* < 0.01, compared with group I; ^d^* p* < 0.01, compared with group II.

### 2.6. Normal Pyramidal Neurons Density

Brain ischemic preconditioning or ischemic tolerance is a phenomenon in which brief episode(s) of sublethal ischemia protect the brain from subsequent, more severe and lethal ischemic insults. The typical ischemic tolerant phenomenon in the organ being preconditioned has been extensively confirmed in different tissues and organs of many species [52,53,54,55,56]. With the progress of the studies on ischemic preconditioning, it has been found that an ischemic preconditioning in an organ remote from the target organ could also protect the target organ from ischemic insult. In rat cerebral ischemia models, a period of ischemia followed by reperfusion causes neuronal degeneration selectively in hippocampal CA1 pyramidal neurons after 48 h of reperfusion but leaves dentate gyrus (DG), CA3, and most cortical neurons intact [57,58,59]. During the 48–72 h delay period, the neurons destined to die look normal under the light microscope. At the ultrastructural level, however, disaggregation of polyribosomes, abnormalities of the Golgi apparatus, deposition of dark substances, and modification of postsynaptic densities have been reported [60,61,62,63,64].

**Figure 1:**
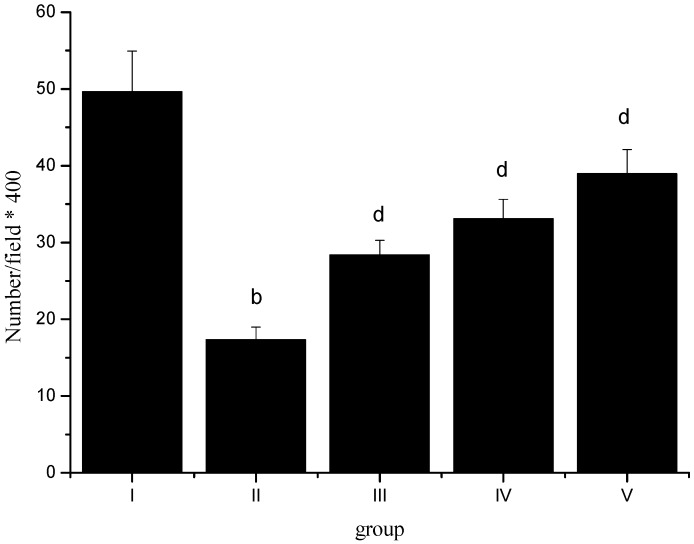
Effect of sevoflurane postconditioning on normal pyramidal neurons density. ^b^* p* < 0.01, compared with group I; ^d^* p* < 0.01, compared with group II.

After CIRI, normal pyramidal neurons number of rats’ cortex in group II (number/field × 400) was significantly reduced (*p* < 0.01). Sevoflurane postconditioning could effectively provent normal pyramidal neurons loss (*p* < 0.01) (Figure 1).

### 2.7. Histopathological Study

Representative histopathological photographs of sections were examined. Representative images of the sham group showed no signs of histopathological abnormalities. In contrast, the images from an animal in the CIRI group exhibited necrotic changes with pronounced vacuolization, intensely eosinophilic cytoplasm, Nissl granule loss, and pyknosis. Sevoflurane postconditioning attenuated CIR-induced histopathological abnormalities. Representative images from the sevoflurane postconditioning group exhibited mild destruction with significantly more normal neurons.

### 2.8. Effect of Sevoflurane Postconditioning on Infarct Volume after Ischemia-Reperfusion

**Figure 2:**
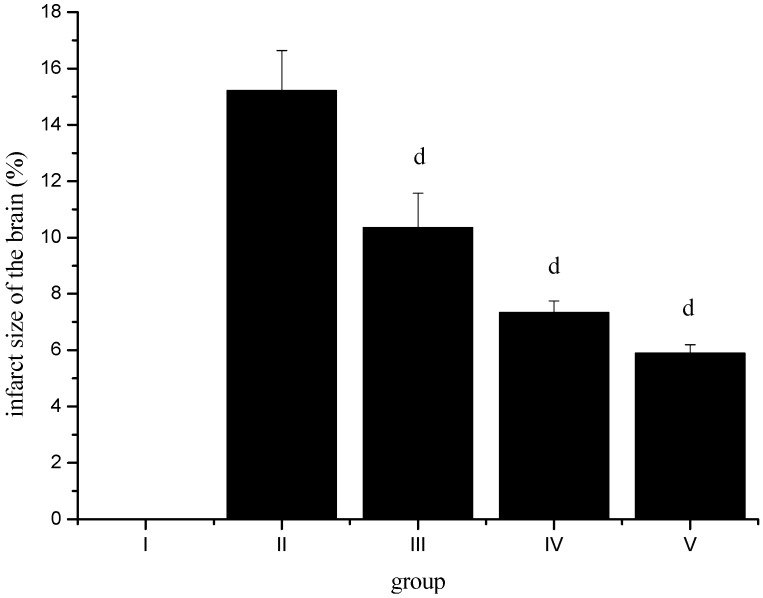
Effect of sevoflurane postconditioning on infarct volume after ischemia-reperfusion. ^d^* p* < 0.01, compared with group II.

In order to further examine the protective effect of sevoflurane postconditioning against an ischemia/reperfusion insult, we measured the infarct volume. As shown in Figure 2, infarct volume was 15.21 ± 1.43% in group II animals, it was significantly reduced in group III-V animals. These observations indicate that sevoflurane postconditioning can degrade ischemia/reperfusion-induced brain injury.

## 3. Experimental

### 3.1. Animals

A total of 40 young-adult, male Wistar rats weighing 240–280 g were used. The rats were housed in groups of three to four in plastic cages (39 cm × 33 cm × 16 cm) at a controlled temperature (22 ± 1 °C) on a 12 h light/dark cycle (lights on at 07:00) with constant air renewal. Food and water were offered ad libitum. These housing conditions were maintained until the end of the experiments.

### 3.2. Experimental Protocol

In the present experiments, rats were placed in an airtight anesthesia chamber and spontaneously breathed O_2_ or sevoflurane during postconditioning. The sevoflurane concentrations in the gases from the outlet of the chambers were monitored with an anesthetic gas analyzer (Dräger). In the experiments, rats were randomly assigned into five separate experimental groups I–V (n = 8 in each group). In the sham group (I), rats were subjected to the same surgery procedures except for occlusion of the middle cerebral artery and exposed to 1.0 MAC sevoflurane 90 min after surgery for 30 min. CIR control rats (group II) were subjected to MCAO for 90 min and exposed to O_2_ for 30 min at the beginning of reperfusion. Sevoflurane 0.5, 1.0 and 1.5 groups (III, IV, V) were all subjected to MCAO for 90 min, but at the beginning of reperfusion exposed to 0.5 MAC, 1.0 MAC or 1.5 MAC sevoflurane for 30 min, respectively.

### 3.3. Middle Cerebral Arterial Occlusion

Briefly, the rats were anesthetized with 10% choral hydrate (350 mg/kg IP), via a midline neck incision, the right common carotid artery (CCA), the external carotid artery (ECA) and the internal carotid artery (ICA) were isolated. A fishing line coated with silicon (diameter 0.32 mm) was introduced into the right CCA and advanced until faint resistance was felt at approximately 18.0 ± 0.5 mm from the carotid bifurcation. After 90 min of occlusion, the suture was removed to allow reperfusion. Rectal temperature was strictly maintained at 37 ± 0.5 °C by warming blanket and lamps from the start of the surgery until the animal recovered from anesthesia. 

Brain was excised, rinsed in ice-cold saline and placed in liquid nitrogen and stored −70 °C for later homogenization and biochemical determinations. Blood was collected for the estimation of NO, NOS, TNF-α, IL-10, IL-1β, TC, TG, LDL-c and HDL-c concentrations.

### 3.4. Biochemical Analysis

TNF-α, IL-10, IL-1β, NO, NOS, TC, TG, LDL-c and HDL-c levels were measured by using ELISA kits according to the manufacturer’s instruction (Genzyme Corp., Cambridge, MA, USA).

The blood and tissue MDA level was determined based on pink color formation with thiobarbituric acid (TBA) at 532 nm [65]. Results were expressed as nmol per gram wet tissue, according to the standard graphic prepared from measurements with a standard solution. 

Blood and tissue GSH concentrations were measured according to the method of Beutler *et al.* [66], using metaphosphoric acid for protein precipitation and 5′-5′-dithio-bis-2-nitrobenzoic acid for color development.

Blood and tissue SOD activity was determined according to the method by Sun *et al*. [67]. One unit of SOD was defined as the amount causing 50% inhibition in the nitroblue tetrazolium (NBT) reduction rate. The SOD activity is expressed as U mg^−1^ protein.

Blood and tissue glutathione peroxidase activity was measured by the method of Paglia and Valentine [68]. GSH-Px activity was detected by decreased absorbance at 340 nm during the conversion of NADPH to NADP^+^.

Blood and tissue catalase activity was measured according to the Aebi method [69], through the observation of hydrogen peroxide (H_2_O_2_) destroyed by the enzyme at 240 nm wavelength in a spectrophotometer.

GR is a ubiquitous enzyme, which catalyzes the reduction of oxidized glutathione (GSSG) to GSH. This assay is based on the oxidation of NADPH to nicotinamide adenine dinucleotide phosphate (NADP), which is catalyzed by limiting a concentration of GR. One GR activity unit is defined as the amount of enzyme catalyzing the reduction of 1 μmole of GSSG per minute at pH 7.6 and 25 °C. One molecule of NADPH is consumed for each molecule of GSSG reduced. Therefore, the reduction of GSSG was determined indirectly by the measurement of the consumption of NADPH, demonstrating decreases in absorbance at 340 nm (A340) as a function of time [70]. 

### 3.5. Histological Study

Twenty-four hours after CIRI injury, eight rats from each group were decapitated and brains were quickly removed. Then, the coronal sections was removed immediately, immersed in 10% formaldehyde and stored at 4 °C for 48 h. After dehydration in graded ethanol, the spinal cord specimens were embedded in paraffin. Coronal sections between optic chiasm and infundibular stalk were cut at a thickness of 5 μm and stained with hematoxylin and eosin (H&E) for evaluation of structural changes. Pyramidal neurons were observed under light microscopy and counted to evaluate loss of normal pyramidal neurons after CIRI and protective effect of sevoflurane.

### 3.6. Measurement of Infarct Volume

Infarct volume was assessed using 2,3,5-triphenyltetrazolium chloride (TTC; Sigma, St. Louis, MO, USA) staining. Three days after reperfusion, rats were injected with 120 mg of pentobarbital. The brain was then removed and cut into 2 mm sections. The slices were placed in a petridish containing 0.05% TTC for 30 min at 37 °C, and periodically agitated to ensure that no slices were resting on the bottom, and then put into 10% formaldehyde. Lesion volumes were calculated from summed, measured areas (Sigma Scan Pro, SPSS software) of unstained tissue in mm^2^ multiplied by the 2-mm slice thickness. The individual measuring the infarct size was blinded as to whether vehicle or morroniside had been administered.

### 3.7. Statistical Analysis

The data were analyzed with SPSS 9.0 for Windows (SPSS Inc., Chicago, IL, USA) by using one-way analyses of variance (ANOVA). Differences between means were determined using Duncan’s multiple range test in which the significance level was defined as *p* < 0.05. 

## 4. Conclusions

In our experiments, sevoflurane postconditioning demonstrated high antioxidant and immunity activities in cerebral ischemia reperfusion rats. On the whole, the complex research of oxidative stress on the brain using the model described gives an opportunity to estimate possible therapeutic effects on the brain using a number of novel perspective antioxidants.

## References

[1] Dröge W. (2002). Free radicals in the physiological control of cell function. Physiol. Rev..

[2] Upham B.L., Wagner J.G. (2001). Toxicant-induced oxidative stress in cancer. Toxicol. Sci..

[3] Dusting G.J., Triggle C. (2005). Are we over oxidized? Oxidative stress, cardiovascular disease, and the future of intervention studies with antioxidants. Vasc. Health Risk Manag..

[4] Harrison D.G., Gongora M.C. (2009). Oxidative stress and hypertension. Med. Clin. N. Am..

[5] Ozsoy N., Can A., Yanardag R., Akev N. (2008). Antioxidant activity of *Smilax excelsa* L. leaf extracts. Food Chem..

[6] Mies G., Kohno K., Hossman K. (1993). MK-801, a glutamate antagonist, lowers flow threshold for inhibition of protein synthesis after middle cerebral artery occlusion of rat. Neurosci. Lett..

[7] Hossman K. (1994). Viability thresholds and the penumbra of focal ischemia. Ann. Neurol..

[8] Chagnac-Amitai Y., Connors B.W. (1989). Horizontal spread of synchronized activity inneocortex by GABA-mediated inhibition. J. Neurophysiol..

[9] Traystman R.J., Kirsch J.R., Koehler R.C. (1991). Oxygen radical mechanisms of brain injury following ischemia and reperfusion. J. Appl. Physiol..

[10] Paradis E., Clavel S., Julien P., Murthy M.R.V., de Bilbao F., Arsenijevic D., Giannakopoulos P., Vallet P., Richard D. (2004). Lipoprotein lipase and endothelial lipase expression in mouse brain: Regional distribution and selective induction following kainic acid-induced lesion and focal cerebral ischemia. Neurobiology.

[11] Bolwell G.P., Davies D.R., Gerrish C., Auh C.K., Murphy T.M. (1998). Comparative biochemistry of the oxidative burst produced by rose and French bean cells reveals two distinct mechanisms. Plant Physiol..

[12] Bolwell G.P., Davies D.R., Gerrish C., Auh C.K., Murphy T.M. (1998). Comparative biochemistry of the oxidative burst produced by rose and French bean cells reveals two distinct mechanisms. Plant Physiol..

[13] Wang H.Y., Wang G.L., Yu Y.H., Wang Y. (2009). The role of phosphoinositide-3-kinase/Akt pathway in propofol-induced postconditioning against focal cerebral ischemia-reperfusion injury in rats. Brain Res..

[14] Young Y., Menon D.K., Tisavipat N., Matta B.F., Jones J.G. (1997). Propofol neuroprotection in a rat model of ischaemia reperfusion injury. Eur. J. Anaesthesiol..

[15] Maier C., Steinberg G.K., Sun G.H., Zhi G.T., Maze M. (1993). Neuroprotection by the alpha 2-adrenoreceptor agonist dexmedetomidine in a focal model of cerebral ischemia. Anesthesiology.

[16] Rajakumaraswamy N., Ma D., Hossain M., Sanders R.D., Franks N.P., Maze M. (2006). Neuroprotective interaction produced by xenon and dexmedetomidine on *in vitro* and *in vivo* neuronal injury models. Neurosci. Lett..

[17] Luo Y., Ma D., Ieong E., Sanders R.D., Yu B., Hossain M., Maze M. (2008). Xenon and sevoflurane protect against brain injury in a neonatal asphyxia model. Anesthesiology.

[18] Sakai H., Sheng H., Yates R.B., Ishida K., Pearlstein R.D., Warner D.S. (2007). Isoflurane provides long-term protection against focal cerebral ischemia in the rat. Anesthesiology.

[19] Yu L.-N., Yu J., Zhang F.-J., Yang M.-J., Ding T.-T., Wang J.-K., He W., Fang T., Chen G., Yan M. (2010). Sevoflurane postconditioning reduces myocardial reperfusion injury in rat isolated hearts via activation of PI3K/Akt signaling and modulation of Bcl-2 family proteins. J. Zhejiang Univ. Sci. B.

[20] Payne R.S., Akca O., Roewer N., Schurr A., Kehl F. (2005). Sevoflurane-induced preconditioning protects against cerebral ischemic neuronal damage in rats. Brain Res..

[21] Yao Y.-T., Fang N.-X., Shi C.-X., Li L.-H. (2010). Sevoflurane postconditioning protects isolated rat hearts against ischemia-reperfusion injury. Chin. Med. J..

[22] Zhang M.-K., Shen S.-Q., Liu R.-Z., Xu F.-H., Chen X.-Q., Li Y.-S., Chen T-T. (2011). Effects of naoshuning on the plasma TNF-α, IL-1β and ICAM-1 levels in a rat brain ischemia-reperfusion injury model. Sci. Res. Essays.

[23] Tomimoto H., Akiguchi I., Wakita H., Kinoshita A., Ikemoto A., Nakamura S., Kimura J. (1996). Glial expression of cytokines in the brains of cerebrovascular disease patients. Acta Neuropathol. (Berlin).

[24] Sairanen T., Carpén O., Karjalainen-Lindsberg M.L., Paetau A., Turpeinen U., Kaste M., Lindsberg P.J. (2001). Evolution of cerebral tumor necrosis factor-alpha production during human ischemic stroke. Stroke.

[25] Carlstedt F., Lind L., Lindahl B. (1997). Proinflammatory cytokines, measured in a mixed population on arrival in the emergency department, are related to mortality and severity of disease. J. Int. Med..

[26] Vila N., Castillo J., Dávalos A., Chamorro A. (2000). Proinflammatory cytokines and early neurological worsening in ischemic stroke. Stroke.

[27] Zaremba J., Skrobanski P., Losy J. (2001). Tumor necrosis factor-alpha is increased in the cerebrospinal fluid and serum of ischaemic stroke patients and correlates with the volume of evolving brain infarct. Biomed. Pharmacother..

[28] Sairanen T.R., Lindsberg P.J., Brenner M., Carpén O., Sirén A.L. (2001). Differential cellular expression of tumor necrosis factor-α and type I tumor necrosis factor receptor after transient global forebrain ischemia. J. Neurol. Sci..

[29] Saito K., Suyama K., Nishida K., Sei Y., Basile A.S. (1996). Early increases in TNF-α, IL-6, and IL-1β levels following transient cerebral ischemia in gerbil brain. Neurosci. Lett..

[30] Liu Y., Jacobowitz D.M., Barone F., McCarron R., Spatz M., Feuerstein G., Hallenbeck J.M., Siren A.L. (1994). Quantitation of perivascular monocytes and macrophages around cerebral blood vessels of hypertensive and aged rats. J. Cereb. Blood Flow Metab..

[31] McCoy M.K., Tansey M.G. (2008). TNF signaling inhibition in the CNS: Implications for normal brain function and neurodegenerative disease. J. Neuroinflamm..

[32] Toda N., Ayajiki K., Okamura T. (2009). Cerebral blood flow regulation by nitric oxide: Recent advances. Pharmacol. Rev..

[33] Vestergaard S., Loft S., Møller P. (2002). Role of inducible nitrogen oxide synthase in benzene-induced oxidative DNA damage in the bone marrow of mice. Free Radic. Biol. Med..

[34] Lipton P. (1999). Ischemic cell death in brain neurons. Physiol. Rev..

[35] Traystman R.J., Kirsch J.R., Koehler R.C. (1991). Oxygen radical mechanism of brain injury following ischemia and reperfusion. J. Appl. Physiol..

[36] Wang Y.-C., Zhang S., Du T.-Y., Wang B., Sun X.-Q. (2010). Hyperbaric oxygen preconditioning reduces ischemia-reperfusion injury by stimulating autophagy in neurocyte. Brain Res..

[37] Chan P.H. (2001). Reactive oxygen radicals in signaling and damage in the ischemic brain. J. Cereb. Blood Flow Metab..

[38] Oh S., Betz A.L. (1991). Interaction between free radicals and excitatory amino acids in the formation of ischemic brain edema in rats. Stroke.

[39] Watson B.D., Busto R., Goldberg W.J., Santiso M., Yoshida S., Ginsberg M.D. (1984). Lipid peroxidation *in vivo* induced reversible global ischemia in rat brain. J. Neurochem..

[40] Evans P.H. (1993). Free radicals in brain metabolism and pathology. Br. Med. Bull..

[41] Candelario-Jalil E., Alvarez D., Merino N., León O.S. (2003). Delayed treatment with nimesulide reduces measures of oxidative stress following global ischemic brain injury in gerbils. Neurosci. Res..

[42] Ozkul A., Akyol A., Yenisey C., Arpaci E., Kiylioglu N., Tataroglu C. (2007). Oxidative stress in acute ischemic stroke. J. Clin. Neurosci..

[43] Ross D. (1988). Glutathione, free radicals and chemotherapeutic agents. Pharmacol. Ther..

[44] Gilgun-Sherki Y., Melamed E., Offen D. (2001). Oxidative stress induced-neurodegenerative diseases: The need for antioxidants that penetrate the blood brain barrier. Neuropharmacology.

[45] Wu G., Fang Y.Z., Yang S., Lupton J.R., Turner N.D. (2004). Glutathione metabolism and its implications for health. J. Nutr..

[46] Cuevas P., Carceller-Benito F., Reimers D. (1989). Administration of bovine superoxide dismutase prevents sequelae of spinal cord ischemia in the rabbit. Anat. Embryol..

[47] Cuevas P., Reimers D., Carceller F., Iimenez A. (1990). Ischemic reperfusion injury in rabbit spinal cord. Protective effect of superoxide dismutase on neurological recovery and spinal infarction. Acta Anat..

[48] Lim K.H., Connolly M., Rose D., Siegman F., Jacobowitz I., Acinapura A., Cunningham J.N. (1986). Ischemic reperfusion injury in rabbit spinal cord. Protective effect of superoxide dismutase on neurological recovery and spinal infarction. Ann. Thorac. Surg..

[49] Taoka Y., Naruo M., Koyanagi E., Urakado M., Inoue M. (1995). Superoxide radicals play important roles in the pathogenesis of spinal cord injury. Paraplegia.

[50] Shah Z.A., Gilani R.A., Sharma P., Vohora S.B. (2005). Cerebroprotective effect of Korean ginseng tea against global and focal models of ischemia in rats. J. Ethno. Pharmacol..

[51] Li J., Han C.F., Li X.X., Du Y., Yang W.Q. (2011). The effect of sevoflurane postconditioning on antioxidation of rat hearts *in vivo*. Chin. Med. Herald.

[52] Kilian J.G., Nakhla S., Griffith K., Harmer J., Skilton M., Celermajer D.S. (2005). Reperfusion injury in the human forearm is mild and not attenuated by short-term ischaemic preconditioning. Clin. Exp. Pharmacol. Physiol..

[53] Badhwar A., Bihari A., Dungey A.A., Scott J.R., Albion C.D., Forbes T.L., Harris K.A., Potter R.F. (2004). Protective mechanisms during ischemic tolerance in skeletal muscle. Free Radic. Biol. Med..

[54] Bedirli A., Kerem M., Pasaoglu H., Erdem O., Ofluoglu E., Sakrak O. (2005). Effects of ischemic preconditioning on regenerative capacity of hepatocyte in the ischemically damaged rat livers. J. Surg. Res..

[55] Glantz L., Avramovich A., Trembovler V., Gurvitz V., Kohen R., Eidelman L.A., Shohami E. (2005). Ischemic preconditioning increases antioxidants in the brain and peripheral organs after cerebral ischemia. Exp. Neurol..

[56] Sileri P., Sica G., Gentileschi P., Venza M., Manzelli A., Palmieri G., Spagnoli L.G., Testa G., Benedetti E., Gaspari A.L. (2004). Ischemic preconditioning protects intestine from prolonged ischemia. Transplant. Proc..

[57] Kirino T. (1982). Delayed neuronal death in the gerbil hippocampus following ischemia. Brain Res..

[58] Pulsinelli W.A., Brierley J.B., Plum F. (1982). Temporal profile of neuronal damage in a model of transient forebrain ischemia. Ann. Neurol..

[59] Smith M.L., Bendek G., Dahlgren N., Rosen I., Wieloch T., Siesjö B.K. (1984). Models for studying long-term recovery following forebrain ischemia in the rat. A 2-vessel occlusion model. Acta Neurol. Scand..

[60] Kirino T., Tamura A., Sato K. (1984). Delayed neuronal death in the rat hippocampus following transient forebrain ischemia. Acta Neuropathol..

[61] Petito C.K., Pusinelli W.A. (1984). Delayed neuronal recovery and neuronal death in rat hippocampus following severe cerebral ischemia: Possible relationship to abnormalities in neuronal processes. J. Cereb. Blood Flow Metab..

[62] Rafols J.A., Daya A.M., O’Neil B.J., Krause G.C., Neumar R.W., White B.C. (1995). Global brain ischemia and reperfusion: Golgi apparatus ultrastructure in neurons selectively vulnerable to death. Acta Neuropathol..

[63] Hu B.R., Park M., Martone M.E., Fischer W.H., Ellisman M.H., Zivin J.A. (1998). Assembly of proteins to postsynaptic densities after transient cerebral ischemia. Neuroscience.

[64] Martone M.E., Jones Y.Z., Young S.J., Ellisman M.H., Zivin J.A., Hu B.R. (1999). Modification of postsynaptic densities after transient cerebral ischemia: A quantitative and three-dimensional ultrastructural study. Neuroscience.

[65] Ohkawa H., Ohishi N., Yagi K. (1979). Assay for lipid peroxides in animal tissues by thiobarbituric acid reaction. Anal. Biochem..

[66] Beutler E., Duran O., Kelly M.B. (1963). Improved method for the determination of blood glutathione. J. Lab. Clin. Med..

[67] Sun Y., Oberley L.W., Li Y. (1988). A simple method for clinical assay of superoxide dismutase. Clin. Chem..

[68] Paglia D.E., Valentine W.N. (1967). Studies on the quantitative and qualitative characterization of erythrocyte glutathione peroxidase. J. Lab. Clin. Med..

[69] Aebi H. (1984). Catalase *in vitro*. Method. Enzymol..

[70] Carlberg I., Mannervik B. (1985). Glutathione reductase. Method. Enzymol..

